# The iron transporter Transferrin 1 mediates homeostasis of the endosymbiotic relationship between *Drosophila melanogaster* and *Spiroplasma poulsonii*

**DOI:** 10.1093/femsml/uqab008

**Published:** 2021-06-25

**Authors:** Alice Marra, Florent Masson, Bruno Lemaitre

**Affiliations:** Global Health Institute, School of Life Sciences, École Polytechnique Fédérale de Lausanne (EPFL), Lausanne, Switzerland; Global Health Institute, School of Life Sciences, École Polytechnique Fédérale de Lausanne (EPFL), Lausanne, Switzerland; Global Health Institute, School of Life Sciences, École Polytechnique Fédérale de Lausanne (EPFL), Lausanne, Switzerland

**Keywords:** endosymbiosis, *Spiroplasma*, transferrin, iron sequestration

## Abstract

Iron is involved in numerous biological processes in both prokaryotes and eukaryotes and is therefore subject to a tug-of-war between host and microbes upon pathogenic infections. In the fruit fly *Drosophila melanogaster*, the iron transporter Transferrin 1 (Tsf1) mediates iron relocation from the hemolymph to the fat body upon infection as part of the nutritional immune response. The sequestration of iron in the fat body renders it less available for pathogens, hence limiting their proliferation and enhancing the host ability to fight the infection. Here we investigate the interaction between host iron homeostasis and *Spiroplasma poulsonii*, a facultative, vertically transmitted, endosymbiont of *Drosophila*. This low-pathogenicity bacterium is devoid of cell wall and is able to thrive in the host hemolymph without triggering pathogen-responsive canonical immune pathways. However, hemolymph proteomics revealed an enrichment of Tsf1 in infected flies. We find that *S. poulsonii* induces *tsf1* expression and triggers an iron sequestration response similarly to pathogenic bacteria. We next demonstrate that free iron cannot be used by *Spiroplasma* while Tsf1-bound iron promotes bacterial growth, underlining the adaptation of *Spiroplasma* to the intra-host lifestyle where iron is mostly protein-bound. Our results show that Tsf1 is used both by the fly to sequester iron and by *Spiroplasma* to forage host iron, making it a central protein in endosymbiotic homeostasis.

## Teaser

We show that the iron transporter Tsf1 is a key regulator of Drosophila's interaction with the heritable symbiotic bacteria *Spiroplasma poulsonii*.

## BACKGROUND

The sequestration of trace minerals by a host organism is increasingly recognized as part of a ‘nutritional’ immunity, as it limits microbes’ proliferation (Hennigar and McClung 2016). Iron in particular is involved in numerous processes in both eukaryotes and prokaryotes, and has been identified as a critical trace metal in determining the outcome of pathogenic infections (Nairz *et al*. 2010). Consequently, iron-transporting proteins, which are involved in iron sequestration upon infection, are under strong selective pressure in the arms race between hosts and pathogens (Barber and Elde 2014).

Similarly to mammals, the fruit fly *Drosophila melanogaster* uses iron sequestration as an immune defense mechanism (Iatsenko *et al*. 2020). *Drosophila* iron transport and storage rely on three Ferritin (Fer) and three transferrin homologues (Tsf1, Tsf2 and Tsf3). Ferritins are involved in iron absorption, transport and storage and are particularly important during embryonic development (Missirlis *et al*. 2007; Tang and Zhou 2013; González-Morales *et al*. 2015). More recently, Fer1 has also been identified as a potent antioxidant in mitochondria (Mumbauer *et al*. 2019). *Drosophila* Transferrins are considered as transport proteins based on their homology with mammalian Transferrins, although genetics and expression studies have revealed that the three isoforms have distinct and initially unexpected functions. Tsf2 is an integral component of epithelial septate junctions and may have no role in iron transport (Tiklová *et al*. 2010). Tsf3 is poorly characterized but possibly involved in the circadian rhythm regulation (Mandilaras and Missirlis 2012). Tsf1 participates in iron trafficking (Xiao *et al*. 2019) and is inducible upon infection. Its involvement in nutritional immunity has been experimentally demonstrated: by mediating iron sequestration from the hemolymph to the fat body, it reduces iron availability in the hemolymph and consequently enhances fly resistance to infections by *Pseudomonas aeruginosa* and Mucorales fungi (Iatsenko *et al*. 2020).

Iron management is however not central in pathogenic interactions only: a growing number of cooperative iron management strategies is getting described between host and mutualistic microbes (Chen and Ayres 2020). Insects are particularly concerned with associations with endosymbiotic bacteria, i.e. bacteria living within host tissues (Masson and Lemaitre 2020). Some endosymbionts are intracellular and are obligate for the host, as they provision nutrients without which the host cannot develop properly (Moran and Telang 1998). Others are facultative (they do not systematically infect all individuals of their host species) and provide the host with context-dependent ecological advantages, such as a reduction of predation risk (Polin *et al*. 2015), feeding specialization (Tsuchida, Koga and Fukatsu 2004) or protection against natural enemies (Oliver *et al*. 2003; Scarborough, Ferrari and Godfray 2005; Teixeira, Ferreira and Ashburner 2008; Xie, Vilchez and Mateos 2010; Hamilton and Perlman 2013). Some facultative endosymbionts also evolved the ability to manipulate their host reproduction to enhance their own prevalence in the insect population (Engelstädter and Hurst 2009; Hurst and Frost 2015). As endosymbionts are confined within host tissues, they get supplied with nutrients by carrying on intensive metabolic interactions with their host. These interactions were largely studied for organic molecules such as amino-acids and vitamins (Douglas 2016), but little is known about the function of trace metals in endosymbiotic homeostasis. Proteomics on a free-living relative of the aphid facultative endosymbiont *Serratia symbiotica* revealed several proteins involved in iron metabolism suspected to participate in host iron acquisition at the nascent stage of symbiosis (Renoz *et al*. 2017). The Tsetse fly facultative endosymbiont *Sodalis glossinidius* also expresses genes encoding for iron uptake and transport (Runyen-Janecky *et al*. 2010) and a mutant defective for the outer membrane heme transporter HemR has an impaired ability to colonize the host gut, indicating that iron metabolism is important to sustain the tsetse fly symbiotic homeostasis (Hrusa *et al*. 2015). Iron homeostasis has also been highlighted as a key factor regulating *Wolbachia* interaction with its insect hosts. *Wolbachia* is an extremely widespread endosymbiont that can efficiently manipulate the reproduction of various arthropod hosts by four distinct mechanisms that helped it reach a prevalence of over 80% in some host species (Duron *et al*. 2008; Werren, Baldo and Clark 2008; Medina, Russell and Corbett-Detig 2019). It produces Bacterioferritins that scavenge iron to sustain bacterial proliferation and consequently help the host coping with iron overload in the diet (Kremer *et al*. 2009). *Wolbachia* infection also confers a fecundity benefit to *D. melanogaster* reared on both low-iron and high-iron diets, indicating a global buffering role of *Wolbachia* for this nutrient (Brownlie *et al*. 2009). These examples suggest that iron could be an important nutrient in mediating homeostasis in a wide range of insect endosymbioses.

*Spiroplasma poulsonii* (hereafter *Spiroplasma*) is one of the two heritable endosymbionts that naturally infect *Drosophila* flies, along with *Wolbachia* (Mateos *et al*. 2006). *Spiroplasma* belong to the Mollicutes, a class of bacteria that is devoid of cell wall. As it does not expose peptidoglycan or other known bacteria-associated molecular patterns on its surface, *Spiroplasma* is not expected to be recognized by the fly immune system, although this assumption is put into question by recent results suggesting a basal stimulation of the Toll pathway (Masson *et al*. 2021). In return, host immunity seems to have no effect on *Spiroplasma* proliferation, suggesting that titer control is rather mediated by metabolic processes (Anbutsu and Fukatsu 2010; Herren and Lemaitre 2011). *Spiroplasma* lives free in the host hemolymph and gets vertically transmitted by co-opting the yolk uptake machinery of oocytes (Herren *et al*. 2013). Most *Spiroplasma* strains also cause two remarkable phenotypes in *Drosophila*: male killing, whereby infected male offspring is killed during early embryogenesis by the action of a bacterial toxin (Harumoto and Lemaitre 2018), and protection the host against nematode and parasitoid wasp infections (Xie, Vilchez and Mateos 2010; Xie *et al*. 2014; Hamilton *et al*. 2016; Ballinger and Perlman 2017).

The metabolic interactions between *Spiroplasma* and *Drosophila* are still largely elusive, although previous reports showed that the bacterium relies on host circulating diacyglycerides to proliferate (Herren *et al*. 2014). The requirement for other nutrients (amino-acids, vitamins or trace metals for example) has not been explored yet. The genome of *Spiroplasma* encodes for three ferritin-like genes of which one is induced upon host contact compared to *in vitro* culture, suggesting a role of iron in endosymbiosis (Masson *et al*. 2018). This prompted us to investigate the role of iron homeostasis in the *Drosophila-Spiroplasma* symbiosis. We first show that *Drosophila* generates a nutritional immune response against *Spiroplasma* by sequestering iron. Using flies carrying a loss-of-function allele of *tsf1*, we further demonstrate that *Spiroplasma* growth relies on host Tsf1, and that the bacteria uptake iron when it is Tsf1-complexed, and not when it is free. This indicates an evolved ability of *Spiroplasma* to highjack the Tsf1-related iron transport of the host to get access to iron to sustain its own growth.

## RESULTS

### Tsf1 is enriched in *Spiroplasma-*infected flies

**Figure 1.**
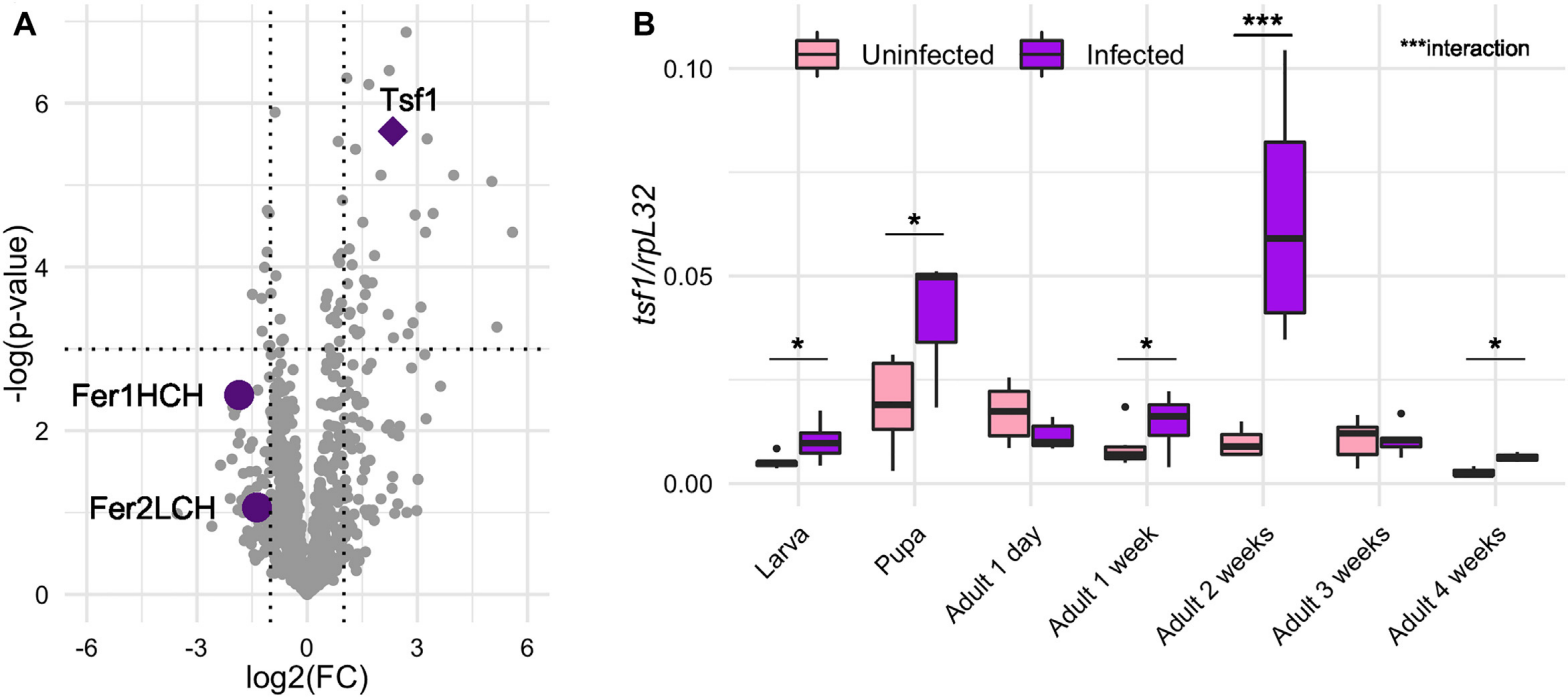
Tsf1 is enriched in *Spiroplasma* infected flies. **(A)** Proteomics profiling of *Spiroplasma*-infected hemolymph from Masson *et al*. (2021). FC represents the fold-change between infected and uninfected flies. Dotted lines indicate the significance thresholds (log2(FC) = −1 or 1 for vertical lines, *P*-value = 0.05 for the horizontal line). Each grey dot represents a protein or a protein group. Purple symbols represent proteins related to iron transport and storage. **(B)** RT-qPCR quantification of *tsf1* transcript levels in infected versus uninfected flies across life stages. Boxplots indicate the mean and interquartile range (IQR). Dots represent values that lie at over 1.5 × IQR from the hinge. Data was analyzed by fitting a linear model with the stage as an ordered factor and the infection status as fixed factor (ANOVA: *P*-value^infection^ = 0.0003; *P*-value^stage^ = 10^−9^; *P*-value^interaction^ = 0.0002) followed by post-hoc Dunnett's pairwise comparisons. *** = *P*-value < 0.001; ** = *P*-value < 0.01; * = *P*-value < 0.05.

A recent proteomics profiling revealed an enrichment of Tsf1 in *Spiroplasma-*infected hemolymph of adult females (Masson *et al*. 2021). Other proteins related to iron transport and storage were identified in the fly hemolymph, notably Fer1HCH and Fer2LCH, but Tsf1 was the only one to be significantly more abundant upon *Spiroplasma* infection (Fig. 1A). Tsf2 and Tsf3 were not detected in the hemolymph.

We first sought to confirm Tsf1 response to *Spiroplasma* infection by measuring the expression of the *tsf1* gene across fly life stages in infected versus uninfected flies (Fig. 1B). Both the infection status and the life stage had a significant effect on *tsf1* expression level (*P*-value Infection = 10^−4^, *P*-value LifeStage = 10^−9^, *P*-value Interaction = 10^−4^). Post-hoc testing indicates that *Tsf1* increased expression upon *Spiroplasma* infection was markedly significant across developmental stages, including in larvae, pupae and adults 1-, 2- and 4-weeks-old (Fig. 1B).

### Host Tsf1 is required for *Spiroplasma* growth but not for vertical transmission

**Figure 2.**
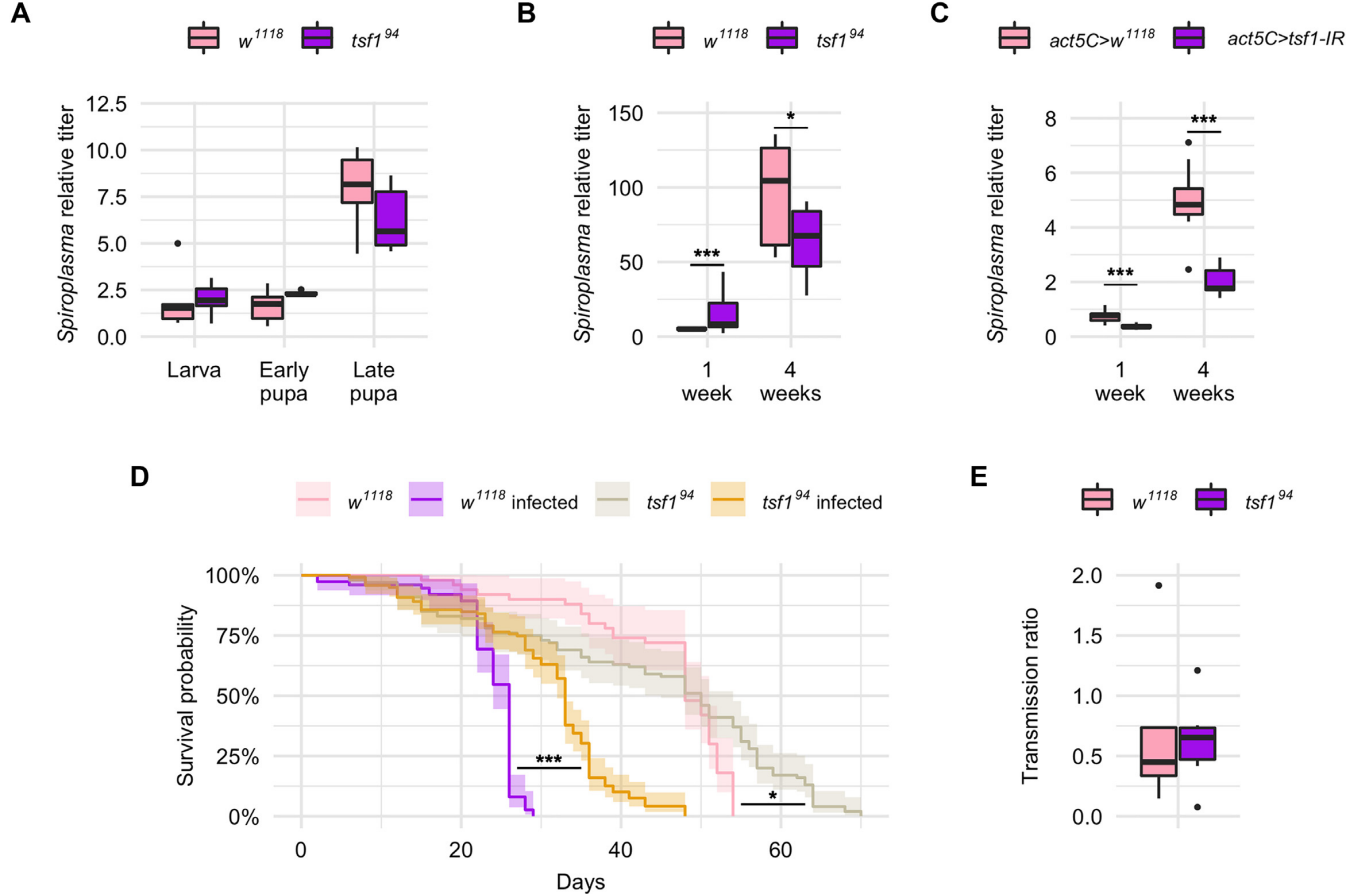
Tsf1 is required for *Spiroplasma* growth at precise life stages of the host. *Spiroplasma* qPCR quantification **(A)** across developmental stages and **(B)** in adult females for *w^1118^* wild-type flies and *tsf1* mutant flies. Data from panels A and B were split for better readability but were analyzed as a whole by fitting a linear model with the stage/age as an ordered factor and the genotype as fixed factor (ANOVA: *P*-value^genotype^ = 0.0357; *P*-value^stage^ = 10^−16^; *P*-value^interaction^ = 0.0003) followed by post-hoc Dunnett's pairwise comparisons. **(C)***Spiroplasma* qPCR quantification in flies expressing a dsRNA targeting *tsf1* under the control of the ubiquitous *act5C-GAL4* driver. *Spiroplasma* relative titer was calculated as the copy number of the *dnaK* gene normalized by that of the host gene *rps17* following the ∆∆CT method. Data was analyzed by fitting a linear model with the age as an ordered factor and the genotype as fixed factor (ANOVA: *P*-value^genotype^ = 10^−8^; *P*-value^age^ = 10^−16^; *P*-value^interaction^ = 0.3625) followed by post-hoc Dunnett's pairwise comparisons. **(D)** Lifespan of infected and uninfected *tsf1* mutant flies compared to their *w^1118^* wild-type counterparts (*N* = 344). Data was analyzed by pairwise Log-Rank test with Benjamini–Hochberg correction for multiple testing. *** = *P*-value < 0.001; * = *P*-value < 0.05. (E) Transmission ratio of *tsf1* mutant flies compared to their *w^1118^* wild-type counterparts. Transmission was calculated as the *Spiroplasma* relative quantification in embryos normalized by the *Spiroplasma* relative quantification in adult females at the time eggs were laid. Data were analyzed with a Student t-test (p-value = 0.8065). Boxplots indicate the mean and interquartile range (IQR). Dots represent values that lie at over 1.5xIQR from the hinge. *** = *P*-value < 0.001; ** = *P*-value < 0.01; * = *P*-value < 0.05.

The observation that *tsf1* was induced by *Spiroplasma* infection, as well as the Tsf1 enrichment in infected hemolymph, pointed to a possible role of Tsf1 and iron in the regulation of the *Drosophila-Spiroplasma* interaction. To test this we infected a mutant fly line carrying a *tsf1* null allele (*tsf1^94^*) (Iatsenko *et al*. 2020) and compared the *Spiroplasma* titer to that of the control *w^1118^* line over all developmental stages of the fly. *tsf1^94^* mutant larvae and pupae exhibited a *Spiroplasma* titer comparable to that of wild-type flies (Fig. 2A). However, the *Spiroplasma* titer was different in the *tsf1^94^* mutant flies compared to the wild-type flies, with a small (although highly significant) increase in 1-week-old flies and a significant decrease in 4-weeks-old flies (Fig. 2B). In wild-type flies, *Spiroplasma* titer increases over fly development and aging: It dramatically increases at mid-pupation and then grows exponentially during adulthood (Herren and Lemaitre 2011). Our results suggest that *tsf1* is required for *Spiroplasma* only at high titer, hence when the most bacteria are competing with the host for iron usage.

Remarkably, the *tsf1^94^*mutant line was unusually difficult to infect by *Spiroplasma* injection and required several attempts before being stably infected. Furthermore, we tried to confirm this phenotype by infecting a stock carrying a chromosomal deficiency lacking the *tsf1* gene, and we could not infect this line despite several attempts. This suggests that the lack of *tsf1* makes flies partially refractory to *Spiroplasma* artificial infections. We could however confirm the requirement of *tsf1* for *Spiroplasma* proper growth using RNA interference (RNAi). A RNAi targeting *tsf1* driven by the ubiquitously expressed driver *actin5C-GAL4* led to a decreased titer in 1-week-old flies and in 4-weeks-old flies, mimicking the phenotype of the *tsf1* mutant in old flies (Fig. 2C).

*Spiroplasma* infection severely reduces *Drosophila* lifespan (Herren *et al*. 2014). The *tsf1^94^* mutant line infected with *Spiroplasma* had an increased lifespan compared to infected controls, in accordance with its decreased bacterial titer (Fig. 2D).

However the *tsf1^94^* mutation did not impair vertical transmission, as evidenced by the stability of the infection over generations and by the titer in embryos that was similar to that of control flies (Fig. 2E). Male-killing was also unaffected by the *tsf1^94^*mutation as the progeny of the mutant line was 100% female for over 2 years after the initial infection.

The decreased titer and consequent increased lifespan of the *tsf1^94^* mutant upon *S. poulsonii* infection was also observed upon *Spiroplasma citri* acute infection (Figure S1, Supporting Information). *Spiroplasma citri* is a closely related species that infects plants and insects with a strict horizontal transmission. It causes lethal infections when injected into the hemolymph of *Drosophila* (Regassa and Gasparich 2006; Herren and Lemaitre 2011). Similar phenotypes between *S. poulsonii* and *S. citri* indicate that *tsf1* requirement might be a requirement shared by several species of the *Spiroplasma* genus, including non-vertically transmitted species, rather than a *S. poulsonii* specificity.

### *Spiroplasma* infection triggers iron sequestration in the fat body

**Figure 3.**
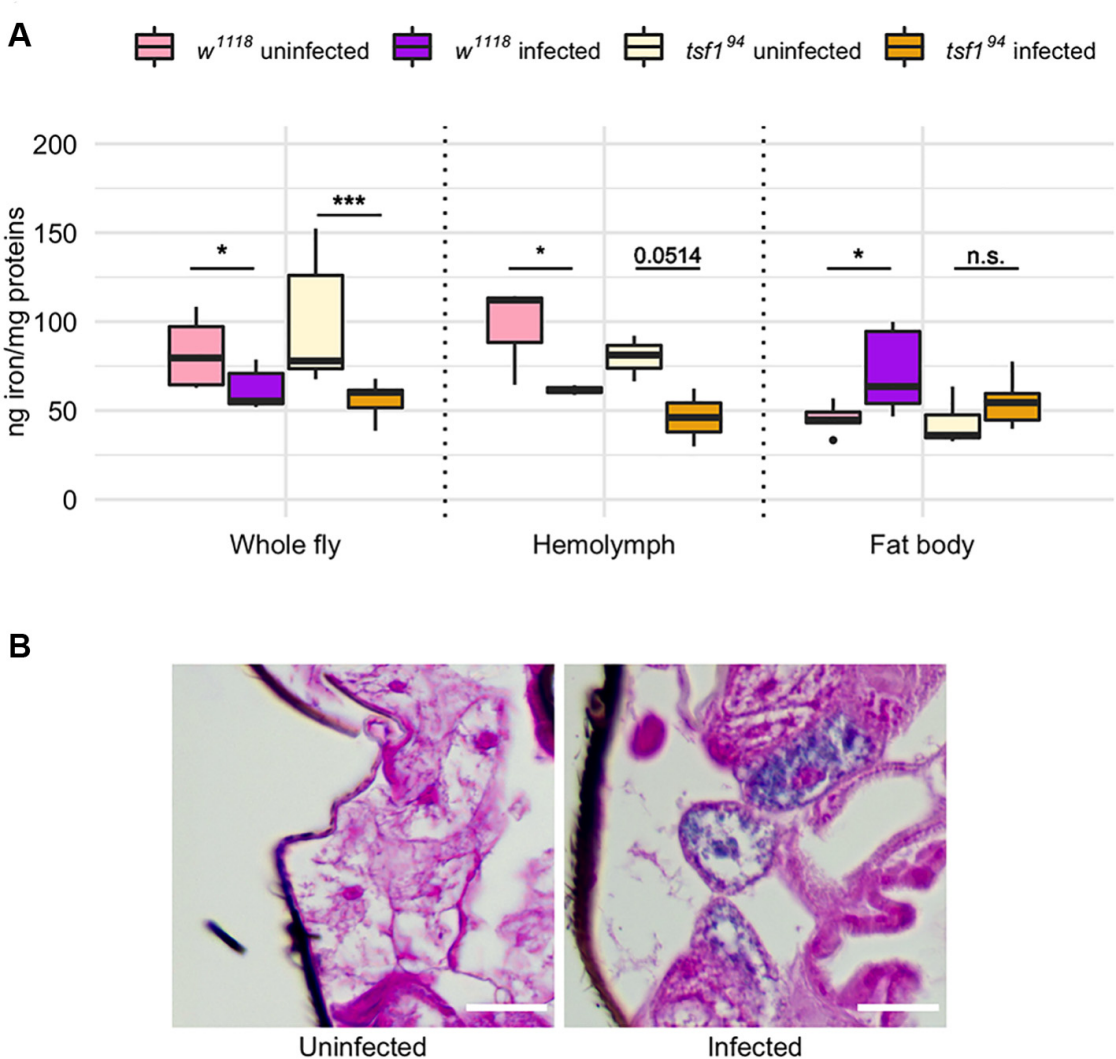
Iron quantification in fly tissues upon *Spiroplasma* infection. **(A)** Iron quantification by ICP-OES in whole flies and in dissected tissues. Iron amount is expressed as ng of iron normalized by mg of proteins in the samples. Data was analyzed by fitting a linear model with the genotype, the infection status and the tissue as fixed factors (ANOVA: *P*-value^genotype^ = 0.3976; *P*-value^infection^ = 0.0070; *P*-value^tissue^ = 0.0003; *P*-value^interaction-genotype*infection^ = 0.1104; *P*-value^interaction-genotype*tissue^ = 0.1652; *P*-value^interaction-tissue*infection^ = 10^−6^) followed by post-hoc Dunnett's pairwise comparisons. Boxplots indicate the mean and interquartile range (IQR). Dots represent values that lie at over 1.5 × IQR from the hinge. *** = *P*-value < 0.001; ** = *P*-value < 0.01; * = *P*-value < 0.05. **(B)** Prussian blue staining on *w^1118^* wild-type flies infected or not by *Spiroplasma*. Iron is visible as blue deposits in the cytosol of adipocytes from infected flies.

Pathogenic infections in *Drosophila* trigger a Tsf1-dependent nutritional immune response whereby iron gets depleted from the hemolymph and sequestrated in the fat body to decrease its availability for invading pathogens (Iatsenko *et al*. 2020). We proceeded with a quantification of iron in fly tissues to see if a similar defense response was observed upon *Spiroplasma* infection, in wild type and *tsf1^94^* mutant flies (Fig. 3A). Iron quantification made on whole flies indicated that *Spiroplasma* causes a significant iron depletion in both genotypes (Fig. 3A). *Spiroplasma-*induced iron depletion was stronger in *tsf1^94^*mutants (−40% in infected flies in average) than in wild type controls (−24% in infected flies in average).

The iron depletion was specific to the hemolymph, where *Spiroplasma* reside. The infection caused a significant (or close to the threshold) iron decrease of similar amplitude in both genotypes. On the other hand, the iron amount in the fat body was tendentially higher in both genotypes. The trend was statistically significant only in the wild type controls for which iron increase in the fat body reached +59% in average upon infection versus only +29% in average in *tsf1^94^* infected mutants (Fig. 3A).

To confirm this results, we then monitored iron sequestration in the fat body by an alternative approach. We stained whole fly sections with Perl's Prussian blue, a stain commonly used to detect iron in tissues. The staining revealed iron inclusions in adipocytes from *Spiroplasma*-infected wild-type flies, seen as blue deposits that were undetectable in the fat body of uninfected flies (Fig. 3B).

Collectively, these results show that *Spiroplasma* induces a nutritional immune response in *Drosophila*. This response translates into iron depletion from the hemolymph and iron sequestration in the fat body. It also indicates that Tsf1 participates in maintaining total iron levels in whole flies and in the iron sequestration in the fat body upon *Spiroplasma* infection, although to a lesser extent compared to the case of an accute infection with pathogenic microbes (Iatsenko *et al*. 2020).

### *Spiroplasma* growth requires Tsf1-complexed iron but not free iron

**Figure 4.**
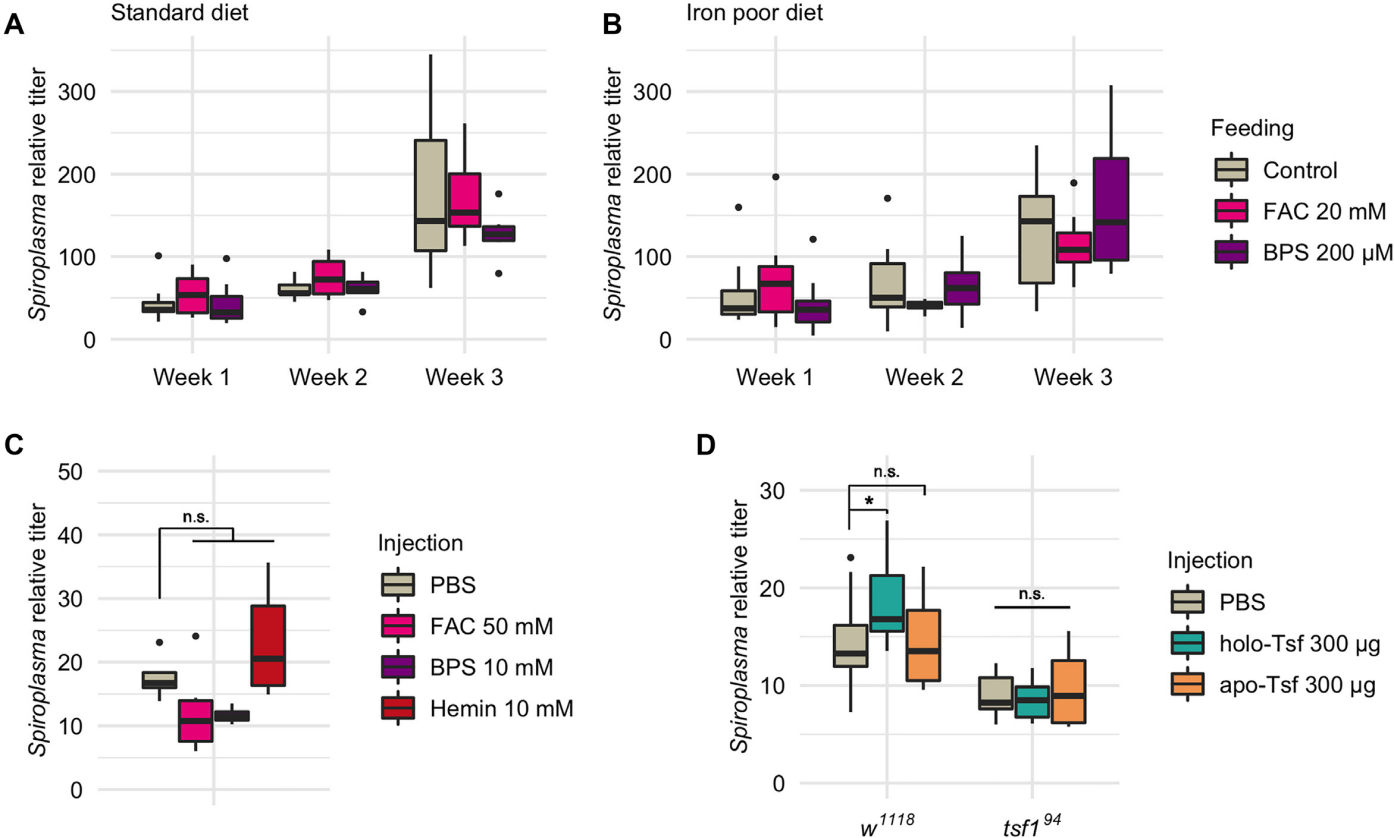
*Spiroplasma* growth is not affected by free iron but fostered by Tsf-complexed iron. *Spiroplasma* quantification in *w^1118^* wild-type flies raised on standard **(A)** or iron poor **(B)** diet supplemented with FAC or BPS. Data was analyzed by fitting a linear model with the age as an ordered factor and the treatment as fixed factor (ANOVA: *P*-value^treatment^ > 0.1; *P*-value^age^ < 0.001; *P*-value^interaction^ > 0.1 for both datasets). **(C)***Spiroplasma* quantification in *w^1118^* flies 1 week after injection of FAC, BPS or hemin. Data was analyzed by fitting a linear model with the treatment as fixed factor (*P*-value^treatment^ > 0.0184) followed by post-hoc Dunnett's pairwise comparisons. **(D)***Spiroplasma* quantification in *w^1118^* wild-type flies or *tsf1* flies 1 week after injection of holo-Tsf (iron bound) or apo-Tsf (not iron bound). Boxplots indicate the mean and interquartile range. Isolated dots represent outliers. *Spiroplasma* relative titer was calculated as the copy number of the *dnaK* gene normalized by that of the host gene *rps17* following the ∆∆CT method. Data was analyzed by fitting a linear model with the genotype and the treatment as fixed factors (ANOVA: *P*-value^genotype^ = 10^−11^; *P*-value^treatment^ = 0.2493; *P*-value^interaction^ = 0.0478) followed by post-hoc Dunnett's pairwise comparisons. Boxplots indicate the mean and interquartile range (IQR). Dots represent values that lie at over 1.5 × IQR from the hinge. *** = *P*-value < 0.001; ** = *P*-value < 0.01; * = *P*-value < 0.05.

The negative impact of Tsf1 absence on *Spiroplasma* growth suggests that *Spiroplasma* relies on this protein to get iron. Therefore we investigated whether *Spiroplasma* growth relies on the uptake of free iron or rather on Tsf1-complexed iron. For this, we compared *Spiroplasma* titer in wild type flies raised on a diet either (i) enriched in iron through ferric ammonium citrate (FAC) supplementation, or (ii) in which iron was made biologically unavailable by adding the iron chelator bathophenanthrolinedisulfonic acid (BPS). FAC and BPS supplementations were performed on a standard diet (Fig. 4A) or on an iron-poor diet (Fig. 4B). We first verified that iron manipulation in the diet had a significant impact on total iron in the flies (Figure S2, Supporting Information), and then performed *Spiroplasma* titer measurements over 3 weeks. None of the treatments (or treatments/time interactions) had a significant effect on *Spiroplasma* titer, which indicates that free iron availability is not required for, or detrimental, to bacterial growth (Fig. 4A and B; Table S1, Supporting Information).

Upon feeding, iron availability for bacteria living in the hemolymph is however dependent on a series of processes: (i) the reduction of free (ferric) iron Fe^3+^ into free (ferrous) iron Fe^2+^ in the gut lumen; (ii) Fe^2+^ uptake in the enterocytes through the metal transporter Malvolio and (iii) iron provisioning to the tissues through Fe-S synthesis in the enterocytes, Fe^3+^ complexation with Ferritin or Tsf1, or direct Fe^3+^ export in the hemolymph through multicopper oxidases (Mandilaras, Pathmanathan and Missirlis 2013; Tang and Zhou 2013; Tang and Zhou 2013). To circumvent all these steps and verify in a more direct fashion the effect of free iron on *Spiroplasma* growth with the least possible bias, we proceeded in injecting FAC or BPS directly in fly thorax and measured *Spiroplasma* titer 1 week later (Fig. 4C). Here again we observed no significant effect, although both treatments led to a slightly lower titer (possibly because of the deleterious effects of the compound injection procedure regardless of their iron-related effect, see Discussion section). We also tried to inject bovine hemin, a porphyrin that contains a ferric ion Fe^3+^. To our surprise, hemin injection caused an upwards trend on *Spiroplasma* titer. The difference was not significant compared to PBS injection, but either significant or close to the threshold when compared to other treatments (*P*-value of the pairwise comparison of Hemin injection with FAC injection = 0.023; with BPS injection = 0.076; Fig. 4C). This could be an indication that iron fosters *Spiroplasma* growth only when complexed to an organic carrier.

We thus wanted to verify if Tsf-complexed iron had a direct positive impact on *Spiroplasma* growth. To this end, we injected commercial mammal Tsf carrying a Fe^3+^ ion (holo-Tsf) or not bound to iron (apo-Tsf) and measured *Spiroplasma* titer after 1 week (Fig. 4D). We observed a significant positive effect on *Spiroplasma* growth upon holo-Tsf injection, but not upon apo-Tsf injection. This provides strong evidence that *Spiroplasma* require iron complexed to Tsf, and not the Tsf protein itself.

Remarkably, the positive effect of holo-Tsf injection was not observed in *tsf1^94^* mutant flies, pointing that iron-protein complexes have an impact only in presence of the native *Drosophila* Tsf1. Iron binding to Transferrins is a spontaneous, competitive, ionic bonding (illustrated by the ability of other metal ions to replace Fe^3+^ upon competition for the binding site (Quarles, Marcus and Brumaghim 2011; Ott, Hartwig and Stillman 2019)), hence ion-exchange from holo- to apo-Tsf are expectable *in vivo*, although this has not been formally demonstrated due to technical hurdles. An attractive hypothesis to explain *Spiroplasma* titer increase upon holo-Tsf injection could be that iron gets transferred from fully loaded bovine Tsf to partially loaded *Drosophila* Tsf1, where it becomes usable by *Spiroplasma*.

Collectively, these results indicate that *Spiroplasma* is not able to benefit from free iron availability but rather relies on iron complexed with host carrier proteins.

## DISCUSSION

Iron is well established as a disputed nutrient between hosts and microbial pathogens. Its function in insect interactions with their microbial symbionts remains however poorly understood. Here, we demonstrate that iron is also disputed between *Drosophila* and its heritable endosymbiont *S. poulsonii*. We show that *Spiroplasma* infection causes an induction of the Tsf1 coding gene in *Drosophila* and a Tsf1 enrichment in the hemolymph of the fly, were *Spiroplasma* resides. By leveraging *Drosophila* genetics, we pinpoint the requirement of *tsf1* gene for a normal *Spiroplasma* growth in older flies. We also show that Tsf1 participates in iron sequestration in the fat body upon *Spiroplasma* infection to a lesser extent than upon pathogenic infections, and that *Spiroplasma* requires Tsf-complexed iron to sustain its growth. Collectively, our results identify Tsf1 as a regulator of endosymbiosis stability, as it is central for both the host iron transport and for symbiont iron uptake.

The requirement of Tsf1 for *Spiroplasma* growth was detectable only in adult flies, and strongest when *Spiroplasma* titer is the highest (Herren and Lemaitre 2011). This result suggests that Tsf1 supply is not limiting bacterial growth when there is a low level of host-symbiont competition for circulating iron. When symbiont demand increases (along with host aging and titer increase), Tsf1 gets undersupplied and does not cover bacterial needs anymore, which hinders *Spiroplasma* growth.

Remarkably, *Spiroplasma* induces *tsf1* expression at earlier stages, when its growth is not yet limited by the *tsf1^94^* mutation. This induction could participate in delaying the moment when Tsf1-bound iron availability becomes limiting for the bacterial. The induction mechanism remains elusive. *tsf1* is induced by the activation of the Toll or the Imd immune pathways upon pathogenic infections (Iatsenko *et al*. 2020). *Spiroplasma* does not activate the Imd pathway, but activates the Toll pathway at a low basal level, possibly through secreted proteases (Masson *et al*. 2021). This basal Toll activation could be sufficient to elicit *tsf1* expression. Alternatively, iron depletion could also be sensed by the fly and trigger *tsf1* expression in an immune-independent manner, although such mechanism has not been described in *Drosophila* so far.

We show that in the case of the chronic, heritable, infection with *Spiroplasma, Drosophila* mounts a nutritional immune response by sequestrating iron. This response resembles that observed in the case of an acute pathogenic infection (Iatsenko *et al*. 2020), with the difference that it is only partially mediated by Tsf1. A conceivable explanation would be that Tsf1 could respond steadily to acute stresses, while other proteins, possibly Tsf3, Ferritins or other unsuspected proteins, would mediate long-lasting sequestration.

Our experiments with FAC and BPS injection also proved intriguing as none of the treatment had a positive impact on *Spiroplasma* growth. The BPS experiment indicates that the bacteria do not directly require free iron. Yet Tsf1 was expected to buffer the FAC supplementation *in vivo*, hence increasing complexed iron availability. An explanation to the lack of positive effect lies in the ability of free iron to produce reactive oxygen species through the Fenton reaction, with deleterious consequences on cells (Galaris and Pantopoulos 2008; Li *et al*. 2016). FAC supplementation positive impact could thus be offset by oxidative stress negative impact, resulting in an overall impaired bacterial growth. On the other hand, hemin or holo-Tsf supplementation could provide iron without triggering the Fenton reaction, hence fostering bacterial growth.

Eventually, we show that holo-Tsf extracted from bovine tissues does not improve *Spiroplasma* growth if the native *Drosophila* Tsf1 is absent (in *tsf1^94^* mutant flies). Combined with the inability of *Spiroplasma* to benefit from free iron supplementation, this is an indication that the bacteria evolved an iron uptake mechanism from *Drosophila* Tsf1 that is protein-specific. The genome sequencing of *S. poulsonii* revealed evolutionary footprints of its adaptation from a free-living to an intra-host lifestyle. An illustrative example is the pseudogenization of its transporter for trehalose (the main circulating sugar in *Drosophila* hemolymph) that is suspected to prevent bacterial overgrowth, hence assuring the long-term stability of the interaction (Paredes *et al*. 2015). We believe the adaptation to Tsf1-complexe iron uptake and not free iron could be another mechanisms coupling *Spiroplasma* growth rate to host metabolism. However, Tsf1 has no known receptor in *Drosophila*, hence the way the protein is internalized by host cells remains elusive, as is the way *Spiroplasma* uptakes iron from it. Further investigation should clarify whether bacterial cells directly internalize the protein, or if iron is scavenged from Tsf1 by means of secreted bacterial proteins.

## MATERIAL AND METHODS

### *Drosophila* and *Spiroplasma* stocks

Standard wild type genotype was *w^1118^* (Bloomington Stock Center BDSC #3605) for all experiments except proteomics, *tsf1* expression measurements and iron feeding experiments for which Oregon-R (BDSC #5) flies were used. The *tsf1^94^* mutant has been published previously (Iatsenko *et al*. 2020) and the UAS-*tsf1*-IR line is publicly available (BDSC #62968). All experiments were carried out with *S. poulsonii* strain Uganda-1 (Ug-1) (Pool, Wong and Aquadro 2006) at 25°C.

### Diet manipulation

Stocks breeding and experiments were carried out on standard cornmeal medium (35.28 g of cornmeal, 35.28 g of inactivated yeast, 3.72 g of agar, 36 mL of fruits juice, 2.9 mL of propionic acid and 15.9 mL of Moldex for 600 mL of medium) at 25°C. Iron poor diet consisted in 10% yeast, 10% sucrose and 0.6% agar (w/v) in water (Chen *et al*. 2016). FAC (Sigma F5879) was supplemented in the diet at 20 mM final concentration and BPS (Sigma 146617, Saint-Louis, MO, USA) at 200 µM final concentration.

### *Spiroplasma* quantification by qPCR

*Spiroplasma* quantifications were performed as previously described (Herren and Lemaitre 2011) using DnaA109F 5′-TTAAGAGCAGTTTCAAAATCGGG-3′ and DnaA246R 5′-TGAAAAAAACAAACAAATTGTTATTACTTC-3′ (Anbutsu and Fukatsu 2003) to quantify *Spiroplasma dnaA* gene and Dmel.rps17F 5′-CACTCCCAGGTGCGTGGTAT-3′ and Dmel.rps17R 5′-GGAGACGGCCGGGACGTAGT-3′ (Osborne *et al*. 2009) to quantify *Drosophila rps17* gene. Relative *Spiroplasma* quantification was calculated as the *dnaA* over *rps17* ratio following the ∆∆CT method (Pfaffl 2001). Quantifications were made in at least 6 biological replicates.

### *tsf1* expression measurement by RTqPCR

*Tsf1* expression was measured by RT-qPCR on pools of 10 individuals at each developmental stage, with six biological replicates or more, as previously described (Iatsenko *et al*. 2020). Primers used were Tsf1-F 5′-GGATCGCCTGCTGAAGAAGA-3′ and Tsf1-R 5′-CCCGGCAGACCAAAGTACTT-3′ for *tsf1* and rpL32-F 5′-GACGCTTCAAGGGACAGTATCTG-3′ and rpL32-R 5′-AAACGCGGTTCTGCATGAG-3′ for the *Drosophila* housekeeping gene *rpL32* (*rp49*) (Romeo and Lemaitre 2008). Relative quantification of *tsf1* transcript was calculated following the ∆∆CT method (Pfaffl 2001).

### Iron measurement using Inductively Coupled Plasma Optical Emission Spectrometry (ICP-OES)

Flies (10 per sample in a least three biological replicates) were used as a whole or their fat body dissected in phosphate buffer saline (PBS). Hemolymph extraction was performed on large batches of flies by centrifugation on filter cartridges as previously described (Iatsenko *et al*. 2020). Sample preparation and iron quantification was then performed as previously described (Iatsenko *et al*. 2020) on a Perkin Elmer Optima 8300 ICP-OES at Laboratoire de Géochimie Environnementale, University of Lausanne, Switzerland. Iron measurements were normalized to the total protein amount in each sample measured using the Pierce BCA Protein Assay Kit (Thermofisher, Waltham, MA, USA).

### Prussian blue staining

Prussian blue staining was performed at the Histology Core Facility of EPFL, Lausanne, Switzerland. Briefly, whole flies were fixed in PBS with 4% paraformaldehyde and 0.1% Triton X-100 overnight and embedded in paraffin in a Sakura VIP6. Sections of 5 µM were stained with using a standard Perl's Prussian blue protocol (Sheehan and Hrapchak 1987) and observed on a Zeiss AxioImager Z1.

### *Spiroplasma citri* challenge

*Spiroplasma citri* infections were performed using the GII3 strain kindly provided by Laure Béven from the UMR1332 ‘Biologie du Fruit et Pathologie,’ INRAE Bordeaux, France (Vignault *et al*. 1980; Saillard *et al*. 2008). *Spiroplasma citri* was grown at 32°C in SP4 medium from 3 days before being injected. A total of 50 µL of culture were centrifuged at 20 000 *g* for 10 min and resuspended in PBS. A total of 9 nL of bacteria suspension or PBS (mock control) were injected in the thorax of young females using a Nanoject II (Drummond) and survival was assessed once a day until all flies died.

### Lifespan assessment

Female flies of each genotype/infection status (*N* = 20 per replicate) were selected within 1 day after eclosion and kept at 25°C on standard cornmeal medium. Medium was changed and survival was assessed three times a week until all flies died. At least three replicates were done for each experimental group.

### Iron derivative injection

An 18 nL injection of each product (or PBS as mock control) was performed in the thorax of young females using a Nanoject II (Drummond). *Spiroplasma* was quantified by qPCR 7 days after the treatment. FAC (Sigma F5879) was injected at 50 mM, BPS (Sigma 146617) at 10 mM and bovine hemin (Sigma H9039) at 10 mM, bovine holo-Tsf (Sigma T1283, Saint-Louis, MO, USA) and human apo-Tsf (Sigma T1147) at 300 µg per fly. *Spiroplasma* quantification was performed 7 days after the treatment in a least four biological replicates.

### Statistical analyses

Analyses were performed using R for macOS (version 3.6.1). Briefly, each dataset was first screened for outliers using the ROUT method (Motulsky and Brown 2006). Seven outliers were discarded in the 1-week-old wild-type group in Fig. 2B, and one was removed from the standard diet, no treatment, 2 weeks-old group in Fig. 4A. *tsf1* expression was analyzed using a linear model with the infection status (uninfected or *Spiroplasma*-infected) as fixed factor and the developmental stage as an ordered factor. *Spiroplasma* titer data in the mutant and RNAi lines were analyzed using a linear model with the genotype as fixed factor and the developmental stage (or adult age) as an ordered factor. Iron quantification data was analyzed by a linear model using genotypes, infection status and tissues as fixed factors. Iron feeding and injection datasets were analyzed using linear models with genotype and/or infection status as fixed factors and age as an ordered factor. Post-hoc pairwise comparisons were carried out with Dunnett's procedure for multiple testing.The normality of residuals of the transmission ratio dataset was verified by Shapiro–Wilks testing, and differences were assessed by a *t*-test.Lifespan assays were analyzed by pairwise Log Rang tests with Benjamini–Hochberg correction for multiple testing.Detailed statistical procedures and results are available in Table S1 (Supporting Information).

## FUNDINGS

This work was supported by the Swiss National Science Foundation grant number 310030_185295.

## AUTHORS’ CONTRIBUTION

AM, FM and BL designed the research. AM performed lab work. AM and FM analyzed data. FM wrote the manuscript, which all co-authors revised and approved.

## Supplementary Material

uqab008_Supplemental_Files
