# Supplementary material for: The iron transporter Transferrin 1 mediates homeostasis of the endosymbiotic relationship between Drosophila melanogaster and Spiroplasma poulsonii
Source: Microlife. 2021 Jun 25;2:uqab008. doi: 10.1093/femsml/uqab008 (PMC10117857; doi:10.1093/femsml/uqab008)
Supplement: uqab008_Supplemental_Files [file uqab008_supplemental_files.zip › TableS1_Statistical_analyses.xlsx.pdf]

The iron transporter Transferrin 1 mediates homeostasis of the endosymbiotic relationship between Drosophila melanogaster and Spiroplasma poulsonii

Statistical analyses

Figure 1 - Tsf1 is enriched in Spiroplasma infected flies (panel B, *tsf1* expression)

Linear model **dCT ~ Genotype * as.ordered(Stage)**

Coefficients:

	Estimate	Std. Error	t value	Pr(>|t|)	Significance
(Intercept)	7.625	0.28806	26.47	< 2e-16	***
Genotype	-0.865	0.40738	-2.123	0.037725	*
as.ordered(Stage)pupa	-1.585	0.40738	-3.891	0.000247	***
as.ordered(Stage)adult1d	-1.66875	0.38107	-4.379	4.67E-05	***
as.ordered(Stage)adult1w	-0.63833	0.40738	-1.567	0.122222	
as.ordered(Stage)adult2w	-0.89	0.45546	-1.954	0.055209	.
as.ordered(Stage)adult3w	-0.871	0.42726	-2.039	0.045764	*
as.ordered(Stage)adult4w	1.01167	0.49893	2.028	0.046899	*
Genotype:as.ordered(Stage)pupa	-0.37833	0.64412	-0.587	0.559093	
Genotype:as.ordered(Stage)adult1d	1.4025	0.53891	2.602	0.011563	*
Genotype:as.ordered(Stage)adult1w	0.09278	0.55159	0.168	0.866974	
Genotype:as.ordered(Stage)adult2w	-1.775	0.64412	-2.756	0.00768	**
Genotype:as.ordered(Stage)adult3w	0.739	0.60424	1.223	0.225949	
Genotype:as.ordered(Stage)adult4w	-0.445	0.7056	-0.631	0.530575	

Residual standard error: 0.7056 on 62 degrees of freedom
Multiple R-squared: 0.6675,
Adjusted R-squared: 0.5978
F-statistic: 9.575 on 13 and 62 DF, p-value: 1.685e-10

ANOVA

Factor	Df	Sum Sq	F value	Pr(>F)	
Genotype	1	7.485	15.033	0.0002579	***
Stage	6	38.765	12.977	1.92E-09	***
Interaction Genotype*Stage	6	15.722	5.2631	0.0001949	***
Residuals	62	30.868			

Dunnett's multiple testing

**Stage = Larva**		
contrast	t.ratio	p.value
Uninfected - Infected	2.123	0.0377
**Stage = Pupa**		
contrast	t.ratio	p.value
Uninfected - Infected	2.492	0.0154
**Stage = Adult 1 day old**		
contrast	t.ratio	p.value
Uninfected - Infected	-1.524	0.1327
**Stage = Adult 1 week old**		
contrast	t.ratio	p.value
Uninfected - Infected	2.077	0.042
**Stage = Adult 2 weeks old**		
contrast	t.ratio	p.value
Uninfected - Infected	5.291	0.0001
**Stage = Adult 3 weeks old**		
contrast	t.ratio	p.value
Uninfected - Infected	0.282	0.7786
**Stage = Adult 4 weeks old**		
contrast	t.ratio	p.value
Uninfected - Infected	2.274	0.0264

Figure 2 - Tsf1 is required for Spiroplasma growth at precise life stages of the host (panels A and B, mutant titer)

Linear model **dCT ~ Genotype * as.ordered(Stage)**

Coefficients:

	Estimate	Std. Error	t value	Pr(>|t|)	
(Intercept)	-0.5974	0.3035	-1.968	0.0521	.
Genotype	-0.2648	0.4293	-0.617	0.5389	
as.ordered(Stage)EarlyPupa	0.0997	0.4293	0.232	0.8169	
as.ordered(Stage)LatePupa	-2.3634	0.4016	-5.886	6.38E-08	***
as.ordered(Stage)Adult1week	-1.779	0.3531	-5.038	2.33E-06	***
as.ordered(Stage)Adult4weeks	-5.9673	0.3774	-15.813	< 2e-16	***
Genotype:as.ordered(Stage)EarlyPupa	-0.4489	0.6071	-0.739	0.4615	
Genotype:as.ordered(Stage)LatePupa	0.6147	0.6033	1.019	0.3109	
Genotype:as.ordered(Stage)Adult1week	-0.7538	0.4897	-1.539	0.1272	
Genotype:as.ordered(Stage)Adult4weeks	0.948	0.5264	1.801	0.075	.

Residual standard error: 0.7435 on 92 degrees of freedom
Multiple R-squared: 0.8802,
Adjusted R-squared: 0.8685
F-statistic: 75.09 on 9 and 92 DF, p-value: < 2.2e-16

ANOVA

Factor	Df	Sum Sq	F value	Pr(>F)	
Genotype	1	2.51	4.5448	0.0356834	*
Stage	4	356.42	161.1719	<2.20E-16	***
Interaction Genotype*Stage	4	12.71	5.7464	0.0003573	***
Residuals	92	50.86			

Dunnett's multiple testing

**Stage = Larva**		
contrast	t.ratio	p.value
w.1118 - tsf1.94	0.617	0.5389
**Stage = Early pupa**		
contrast	t.ratio	p.value
w.1118 - tsf1.94	1.663	0.0998
**Stage = Late pupa**		
contrast	t.ratio	p.value
w.1118 - tsf1.94	-0.825	0.4112
**Stage = Adult 1 week old**		
contrast	t.ratio	p.value
w.1118 - tsf1.94	4.321	0.0001
**Stage = Adult 4 weeks old**		
contrast	t.ratio	p.value
w.1118 - tsf1.94	-2.243	0.0273

Figure 2 - Tsf1 is required for Spiroplasma growth at precise life stages of the host (panel C, RNAi titer)

Linear model **dCT ~ Genotype * as.ordered(Stage)**

Coefficients:

	Estimate	Std.	Error	t value	Pr(>|t|)
(Intercept)	0.485	0.1409	3.443	0.00183	**
genotype	0.9813	0.2053	4.779	5.08E-05	***
as.ordered(Stage)week4	-2.7325	0.2053	-13.308	1.25E-13	***
genotype:as.ordered(Stage)week4	0.2777	0.3	0.926	3.63E-01	

Residual standard error: 0.4226 on 28 degrees of freedom
Multiple R-squared: 0.9277,
Adjusted R-squared: 0.9199
F-statistic: 119.7 on 3 and 28 DF, p-value: 4.473e-16

ANOVA

Factor	Df	Sum Sq	F value	Pr(>F)	
Genotype	1	9.842	55.1156	4.378E-08	***
Stage	1	53.968	302.2237	<2.20E-16	***
Interaction Genotype*Stage	1	0.153	0.8568	0.3625	
Residuals	28	5			

Dunnett's multiple testing

**Stage = Adult 1 week old**		
contrast	t.ratio	p.value
act5C>w.1118 - act5C>tsf1-IR	-4.779	0.0001
**Stage = Adult 4 weeks old**		
contrast	t.ratio	p.value
act5C>w.1118 - act5C>tsf1-IR	-5.756	0.0001

Figure 2 - Tsf1 is required for Spiroplasma growth at precise life stages of the host (panel D, survivals)

Pairwise comparisons using Log-Rank test

p-values	w.1118 uninf.	w.1118 inf.	tsf1.94 uninf.
w.1118 inf.	<2e-16	-	-
tsf1.94 uninf.	0.01	<2e-16	-
tsf1.94 inf.	<2e-16	<2e-16	<2e-16

p-value adjustment method: Benjamini-Hochberg

Figure 2 - Tsf1 is required for Spiroplasma growth at precise life stages of the host (panel E, transmission ratio)

Welch Two Sample t-test

t = 0.25626
df = 5.8658
p-value = 0.8065

Figure 3 - Iron quantification in fly tissues upon Spiroplasma infection.

Linear model **ng_iron_per_mg_proteins ~ genotype*infection*tissue**

Coefficients:

	**Estimate**	**Std. Error**	**t value**	**Pr(>|t|)**	
(Intercept)	81.396	5.883	13.835	<2.00E-16	
genotype	13.379	8.548	1.565	0.122996	
infection	-19.193	9.168	-2.093	0.040704	*
tissue_h	15.609	12.247	1.274	2.08E-01	
tissue_fb	-35.947	10.19	-3.528	8.27E-04	***
genotype:infection	-19.108	12.688	-1.506	0.137494	
genotype:tissue_bh	-30.479	17.431	-1.749	0.085657	.
genotype:tissue_fb	-16.41	13.847	-1.185	0.24081	
infection:tissue_h	-16.294	19.3	-0.844	0.402004	
infection:tissue_fb	46.179	14.239	3.243	0.001962	**
genotype:infection:tissue_h	20.805	27.164	0.766	0.446826	
genotype:infection:tissue_fb	4.524	19.667	0.23	0.818862	

Residual standard error: 18.6 on 58 degrees of freedom
Multiple R-squared: 0.5222
Adjusted R-squared: 0.4316
F-statistic: 5.764 on 11 and 58 DF, p-value: 3.36e-06

ANOVA

**Factor**	**Df**	**Sum Sq**	**F value**	**Pr(>F)**
genotype	1	251.4	0.7262	
infection	1	2711.3	7.833	**
tissue	2	6311.2	9.1166	***
genotype:infection	1	909.7	2.6282	
genotype:tissue	2	1286.2	1.8579	
infection:tissue	2	9849.1	14.2273	***
genotype:infection:tissue	2	203.1	0.2933	
Residuals	58	20075.7		

Dunnett's multiple testing

Genotype = w.1118; tissue = whole fly

contrast	t.ratio	p.value
Uninf. - Inf.	2.093	0.0407

Genotype = tsf1.94 tissue = whole fly

contrast	t.ratio	p.value
Uninf. - Inf.	4.367	0.0001

Genotype = w.1118; tissue = hemolymph

contrast	t.ratio	p.value
Uninf. - Inf.	2.089	0.0411

Genotype = tsf1.94 tissue = hemolymph

contrast	t.ratio	p.value
Uninf. - Inf.	1.99	0.0514

Genotype = w.1118; tissue = fat body

contrast	t.ratio	p.value
Uninf. - Inf.	-2.477	0.0162

Genotype = tsf1.94 tissue = fat body

contrast	t.ratio	p.value
Uninf. - Inf.	-1.198	0.2357

Figure 4 - Spiroplasma growth is not affected by free iron but fostered by Tsf-complexed iron (panel A, iron feeding on standard medium)

Linear model **dCT ~ treatment*as.ordered(time)**

Coefficients:

	**Estimate**	**Std. Error**	**t value**	**Pr(>|t|)**	
(Intercept)	-5.3275	0.21322	-24.986	<2.00E-16	***
treatment_FAC20mM	-0.28964	0.31212	-0.928	0.3574	
treatment_PS100uM	0.11036	0.31212	0.354	0.725	
as.ordered(time)week2	-0.56679	0.31212	-1.816	7.47E-02	.
as.ordered(time)week3	-1.92687	0.30153	-6.39	3.50E-08	***
treatment_FAC20mM:as.ordered(time)week2	0.01768	0.4414	0.04	0.9682	
treatment_PS100uM:as.ordered(time)week2	-0.09893	0.4487	-0.22	0.8263	
treatment_FAC20mM:as.ordered(time)week3	0.18235	0.4511	0.404	0.6876	
treatment_PS100uM:as.ordered(time)week3	0.18045	0.4414	0.409	0.6842	

Residual standard error: 0.6031 on 56 degrees of freedom
Multiple R-squared: 0.6488
Adjusted R-squared: 0.5986
F-statistic: 12.93 on 8 and 56 DF, p-value: 2.457e-10

ANOVA

**Factor**	**Df**	**Sum Sq**	**F value**	**Pr(>F)**	
treatment_f	2	1.393	1.9156	0.1568	
as.ordered(time)	2	36.481	50.1542	3.29E-13	***
treatment_f:as.ordered(time)	4	0.169	0.1164	0.9762	
Residuals	56	20.367			

Figure 4 - Spiroplasma growth is not affected by free iron but fostered by Tsf-complexed iron (panel B, iron feeding on iron-poor medium)

Linear model **dCT ~ treatment*as.ordered(time)**

Coefficients:

	**Estimate**	**Std. Error**	**t value**	**Pr(>|t|)**	
(Intercept)	-5.5031	0.3633	-15.148	<2e-16	***
treatment_FAC20mM	-0.2325	0.5138	-0.453	0.6525	
treatment_PS100uM	0.6237	0.5138	1.214	0.2293	
as.ordered(time)week2	-0.2056	0.5138	-0.4	6.90E-01	
as.ordered(time)week3	-1.2788	0.5138	-2.489	1.55E-02	*
treatment_FAC20mM:as.ordered(time)week2	0.6369	0.7266	0.877	0.3841	
treatment_PS100uM:as.ordered(time)week2	-0.6025	0.7266	-0.829	0.4101	
treatment_FAC20mM:as.ordered(time)week3	0.2451	0.7394	0.331	0.7414	
treatment_PS100uM:as.ordered(time)week3	-1.0481	0.7266	-1.443	0.1542	

Residual standard error: 1.028 on 62 degrees of freedom
Multiple R-squared: 0.3694
Adjusted R-squared: 0.288
F-statistic: 4.539 on 8 and 62 DF, p-value: 0.0002204

ANOVA

**Factor**	**Df**	**Sum Sq**	**F value**	**Pr(>F)**	
treatment	2	0.07	0.0333	0.9672	
as.ordered(time)	2	33.357	15.7972	2.85E-06	***
treatment:as.ordered(time)	4	4.861	1.1511	0.3411	
Residuals	62	65.46			

Figure 4 - Spiroplasma growth is not affected by free iron but fostered by Tsf-complexed iron (panel C, iron injection)

Linear model **dCT ~ treatment**

Coefficients:

	Estimate	Std. Error	t value	Pr(>|t|)	
(Intercept)	-4.1137	0.2636	-15.603	4.22E-11	***
treatmentbfac	0.6679	0.3404	1.962	0.0674	.
treatmentcbps	0.5775	0.3729	1.549	0.141	
treatmentdhemin	-0.3304	0.3404	-0.971	3.46E-01	

Residual standard error: 0.5273 on 16 degrees of freedom
Multiple R-squared: 0.4558
Adjusted R-squared: 0.3538
F-statistic: 4.467 on 3 and 16 DF, p-value: 0.01843

ANOVA

Factor	Df	Sum Sq	F value	Pr(>F)	
treatment	3	3.7261	4.4672	0.01843	*
Residuals	16	4.4486			

Dunnett's multiple testing

contrast	t.ratio	p.value	
PBS - FAC	-1.962	0.2592	
PBS - BPS	-1.549	0.4609	
PBS - Hemin	0.971	0.7933	
FAC - BPS	0.266	0.9943	
FAC - Hemin	3.279	0.023	*
BPS - Hemin	2.667	0.0762	.

Figure 4 - Spiroplasma growth is not affected by free iron but fostered by Tsf-complexed iron (panel D, Tsf injection)

Linear model **dCT ~ treatment*genotype**

Coefficients:

	Estimate	Std. Error	t value	Pr(>|t|)	
(Intercept)	-3.77821	0.11084	-34.087	<2.00E-16	***
treatment_300ugholo	-0.47906	0.1671	-2.867	0.00586	**
treatment_300ugapo	-0.01679	0.23513	-0.071	0.94335	
genotype	0.64384	0.15178	4.242	8.56E-05	***
treatment_300ugholo:genotype	0.55218	0.23023	2.398	1.99E-02	*
treatment_300ugapo:genotype	-0.01884	0.33021	-0.057	0.95471	

Residual standard error: 0.4147 on 55 degrees of freedom
Multiple R-squared: 0.5713
Adjusted R-squared: 0.5324
F-statistic: 14.66 on 5 and 55 DF, p-value: 3.979e-09

ANOVA

Factor	Df	Sum Sq	F value	Pr(>F)	
treatment	2	0.4901	1.4247	0.24933	
genotype	1	10.9803	63.8383	9.06E-11	***
treatment:genotype	2	1.1058	3.2144	0.04784	*
Residuals	55	9.4601			

Dunnett's multiple testing

Genotype = w.1118

contrast	t.ratio	p.value	
PBS - holoTsf	2.867	0.0164	*
PBS - apoTsf	0.071	0.9981	
holoTsf - apoTsf	-1.909	0.1549	

Genotype = tsf1.94

contrast	t.ratio	p.value
PBS - holoTsf	-0.462	0.9115
PBS - apoTsf	0.154	0.9908
holoTsf - apoTsf	0.454	0.9144

Figure S1. *tsf1* mutant phenotype upon *Spiroplasma citri* acute infection (panel A, titer)

Welch Two Sample t-test

t = 4.8166
df = 11.654
p-value = 0.0004576 ***

Figure S1. *tsf1* mutant phenotype upon *Spiroplasma citri* acute infection (panel B, survival)

Pairwise comparisons using Log-Rank test

p-values	w.1118 uninf.	w.1118 inf.	tsf1.94 uninf.
w.1118 inf.	6.9E-12	-	-
tsf1.94 uninf.	0.7316	6.9E-12	-
tsf1.94 inf.	6.9E-12	0.0043	1.3E-11

p-value adjustment method: Benjamini-Hochberg
