# Supplementary figures and images for: The iron transporter Transferrin 1 mediates homeostasis of the endosymbiotic relationship between Drosophila melanogaster and Spiroplasma poulsonii
Source: Microlife. 2021 Jun 25;2:uqab008. doi: 10.1093/femsml/uqab008 (PMC10117857; doi:10.1093/femsml/uqab008)

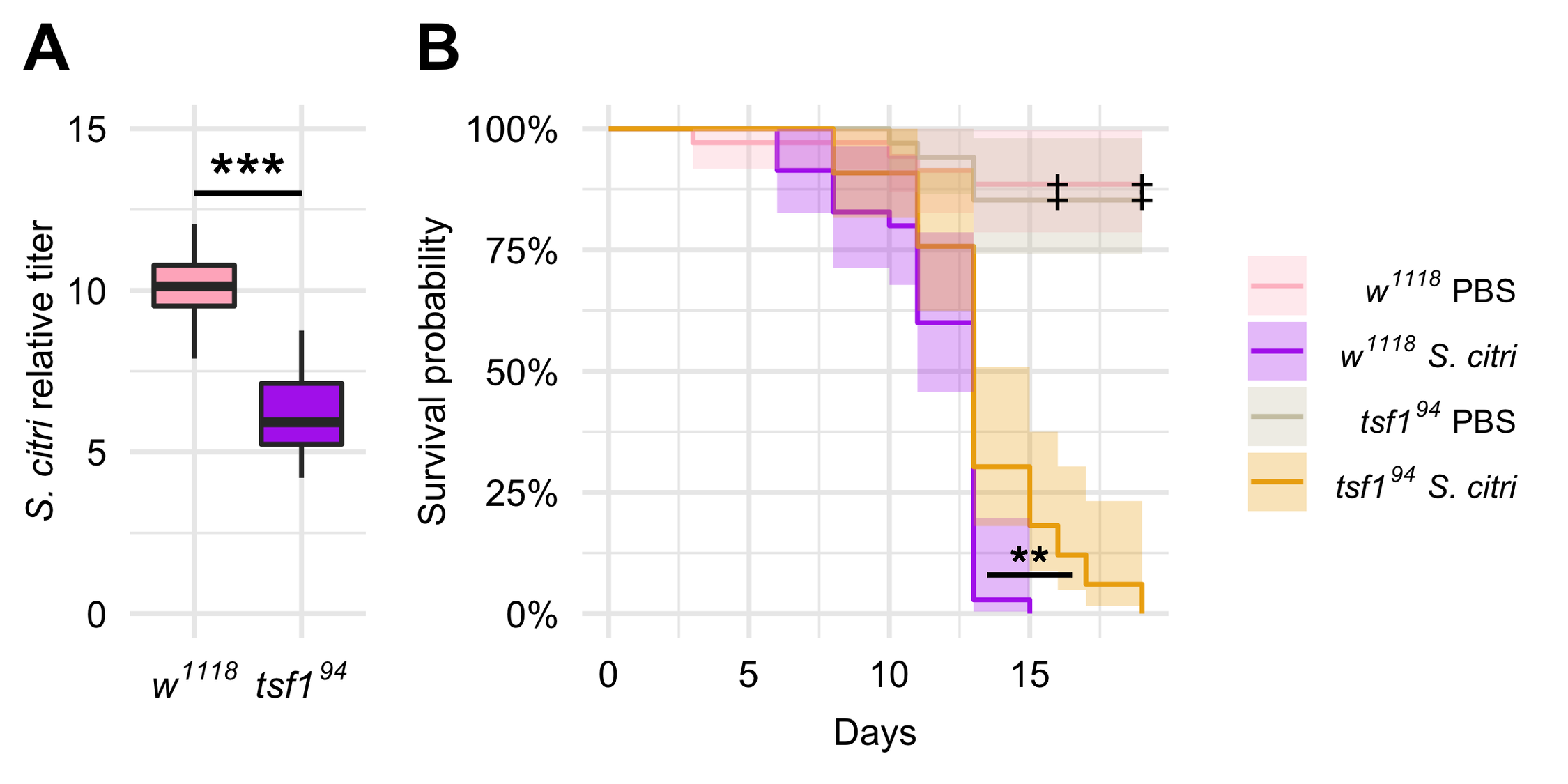

Supplement: uqab008_Supplemental_Files [file uqab008_supplemental_files.zip › figS1.tif]

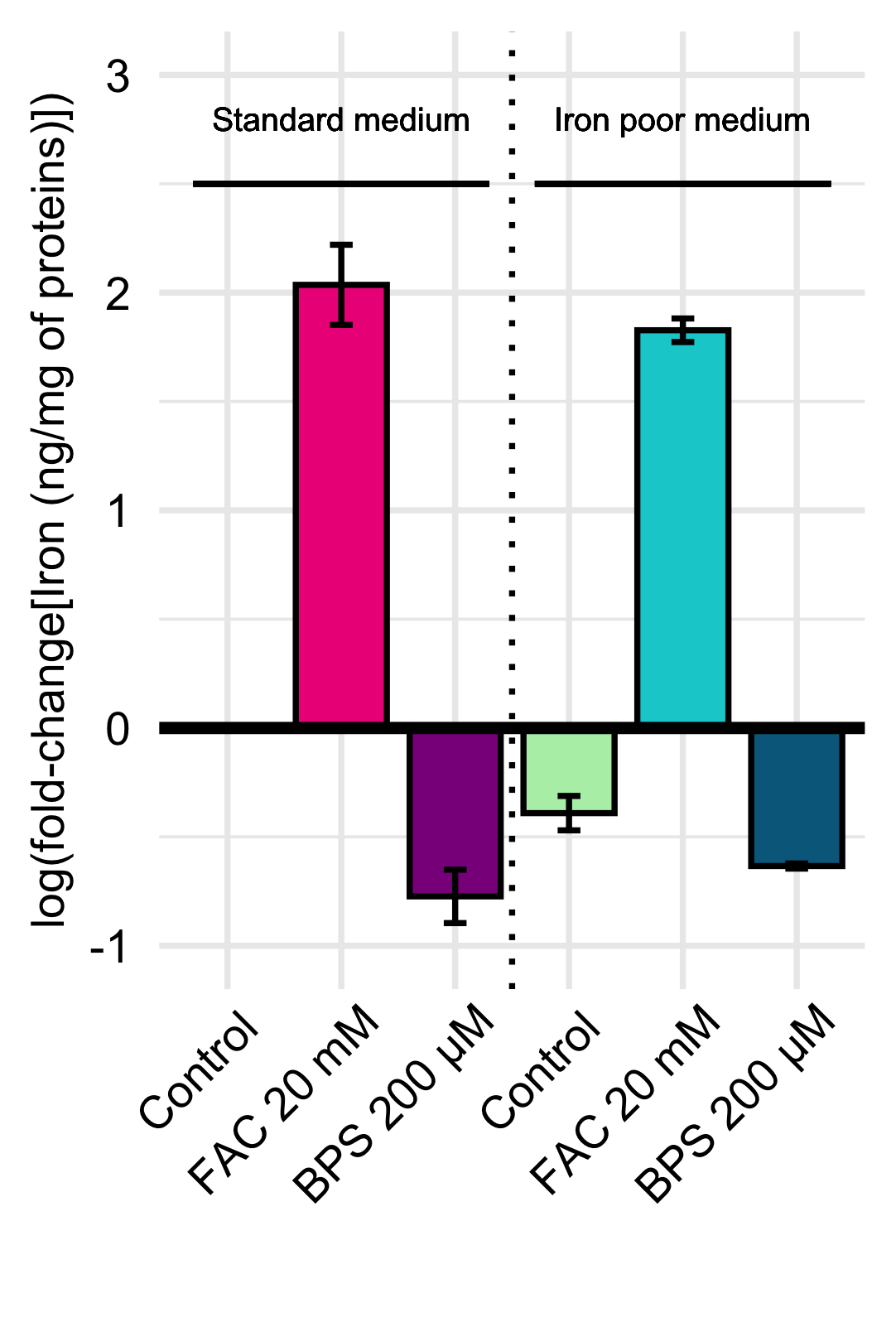

Supplement: uqab008_Supplemental_Files [file uqab008_supplemental_files.zip › figS2-food.tif]
